# Experimental Intrastriatal Applications of Botulinum Neurotoxin-A: A Review

**DOI:** 10.3390/ijms19051392

**Published:** 2018-05-07

**Authors:** Alexander Hawlitschka, Andreas Wree

**Affiliations:** Institute of Anatomy, Rostock University Medical Center, Gertrudenstraße 9, 18057 Rostock, Germany; andreas.wree@med.uni-rostock.de

**Keywords:** botulinum toxin, 6-OHDA, basal ganglia, striatum, Parkinson’s disease, brain, acetylcholine

## Abstract

Parkinson’s disease (PD) is one of the most frequent neurodegenerative disorders. Its main pathophysiological characteristic is the loss of dopaminergic neurons in the substantia nigra pars compacta followed by a lack of striatal dopaminergic input and a consequent disinhibition of tonically active cholinergic interneurons. The resulting striatal hypercholinism causes major motor symptoms in PD. Anticholinergic pharmacotherapies have antiparkinsonian effects on motor symptoms, but, due to systemic actions, also numerous severe side effects occur on a regular basis. To circumvent these side effects, a local anticholinergic therapy acting exclusively in the striatum would be reasonable. Botulinum neurotoxin-A (BoNT-A) is synthesized by *Clostridium botulinum* and blocks the release of acetylcholine from the presynaptic bouton. For several decades, BoNT-A has been used successfully for medical and cosmetic purposes to induce controlled paralyses of single muscles. Our group and others investigated the experimental treatment of striatal hypercholinism by the direct injection of BoNT-A into the striatum of rats and mice as well as of hemiparkinsonian animal models. This review gives an overview of the most important results of the experimental intrastriatal BoNT-A application, with a focus on hemiparkinsonian rats.

## 1. Introduction

### 1.1. Idiopathic Parkinson Syndrome and Current Treatment Concepts

Parkinson’s disease (PD) is the second most frequent neurodegenerative disease. Main motor symptoms in PD are caused by axonal degeneration of dopaminergic fibers in the striatum (caudate-putamen, CPu) and subsequent or parallel loss of dopaminergic neurons in the substantia nigra pars compacta (SNpc) [1,2,3,4]. Up to now, not all mechanisms of axonal and neuronal loss related with idiopathic Parkinson’s syndrome are understood. Axonal degeneration and cell death of dopaminergic neurons in the SNpc leads to the loss of afferent dopaminergic fibers in the CPu. The lack of the neurotransmitter dopamine in the striatum interferes with other transmitters such as glutamate, gamma-aminobutyric acid (GABA) and acetylcholine [5]. PD-related motor symptoms involve slowdown of movements and rigidity and are due to disturbances of the basal ganglia circuitry, especially the direct, indirect and hyperdirect pathways [6].

The direct pathway is disturbed in PD as follows: a cohort of medium spiny neurons (MSNs) in the CPu bears excitatory D_1_ receptors. MSNs project onto the internal globus pallidus (GPi). The PD-associated lack of dopaminergic input into the CPu causes a decreased activity of these GABAergic MSNs and thereby leads to a disinhibition of the GABAergic projection neurons of the GPi. The GPi sends its GABAergic efferences to the ventrolateral thalamic nucleus (VL) and the resulting inhibition is increased in the VL. Thereby, the VL cannot excite the premotor cortex, which in turn generates fewer impulses for motor activity in the contralateral body side [7,8,9].

**Figure 1:**
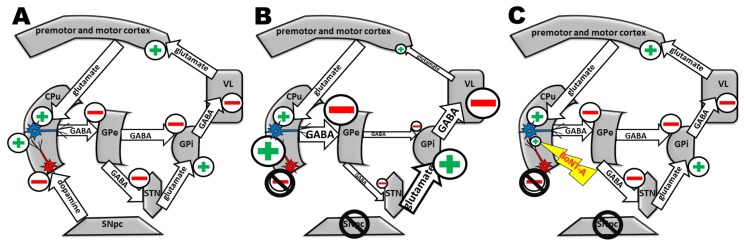
Indirect pathway under normal, parkinsonian and parkinsonian + BoNT-A-treated conditions. Simplified schemes of the indirect pathway of the basal ganglia circuitry. Arrows symbolize directions of connections. Broader arrows show pathologically PD-related increased activity, thinner ones decreased activity. Major transmitters are indicated. A “+” stands for excitation, a “−“ stands for inhibition in the respective compartment. Red neurons in the striatum (CPu) stand for cholinergic interneurons, blue neurons for medium spiny neurons (MSNs) projecting to the external globus pallidus (GPe). (**A**) Indirect loop under normal conditions: dopaminergic efferent fibres of the substantia nigra pars compacta (SNpc) directed to the CPu inhibit the tonically active cholinergic interneurons. The cholinergic interneurons inhibit MSNs, which in turn functionally inhibit the GPe by GABA. The GPe primarily inhibits the subthalamic nucleus (STN), and the STN activates the internal globus pallidus (GPi). Next, via GABA transmission the GPi inhibits the thalamic ventral lateral nucleus (VL). Lastly, VL activates premotor and motor cortices for movement initiation. (**B**) Disturbance of the indirect loop of the basal ganglia circuitry under parkinsonian conditions: the majority of the dopaminergic neurons in the SNpc are degenerated leading to a loss of dopaminergic input into the CPu, resulting in cholinergic interneurons’ hyperactivity with an increased release of acetylcholine. By this, the MSNs become more activated, and, consequently, the GPe more inhibited. This leads to a reduced inhibition of the STN that in turn increasingly activates the GPi. The overactive GPi inhibits strongly the VL. In consequence, the VL insufficiently activates the premotor and motor cortices followed by reduced movement initiation. (**C**) Working hypothesis of intrastriatal BoNT-A application in an animal model of hemi-PD: BoNT-A disrupts the synaptic transmission between cholinergic interneurons and MSNs in the CPu. Thereby, the activity of the MSNs of the indirect loop should return to normal and the following disturbances of this basal ganglia circuitry shall be abrogated. The activity of VL is expected to normalize.

In addition, the indirect pathway is affected in PD: cholinergic interneurons in the striatum bear inhibitory D_2_ receptors and become overactive in PD due to the loss of dopaminergic input. This leads to a hyperactivity of the GABAergic MSNs, which project onto the external globus pallidus (GPe). Consequently, the inhibition of the GPe is increased, whereby its target areas—mainly the subthalamic nucleus (STN)—are disinhibited. Thus, the GPi is intensively stimulated, resulting in strong inhibition of the VL by the GPi. Because of its enhanced inhibition in PD, the premotor cortex is excited less intensely by the VL, which leads to reduced initiations of movements in the contralateral body side [7,8,9] (Figure 1A,B). This mechanism is mainly involved in bradykinesia and akinesia in PD. Although the CPu also gets cholinergic afferences from the pedunculopontine and the laterodorsal tegmental nuclei [10], the overactivity of striatal cholinergic interneurons is responsible for the pathologically increased acetylcholine level in the CPu in PD [11,12,13,14].

To recover the affected parts of the basal ganglia circuitry, a bundle of therapeutic concepts has been developed. The gold standard in the therapy of PD is the enhancement of the pathologically decreased dopamine concentration in the CPu by administration of the dopamine precursor L-3,4-dihydroxyphenylalanine (L-DOPA) [15,16,17,18,19]. To avoid the fast clearance of L-DOPA, catechol-*O*-methyltransferase inhibitors such as entacapon and tolcapon as well as DOPA decarboxylase inhibitors such as carbidopa and benserazide are additionally administered. Thereby, the availability and the passage through the blood–brain barrier should be improved [16,20]. To raise the level of dopamine in the brain by inhibition of its depletion, substances such as selegiline and rasagiline are used, which are mainly considered to work as irreversible monoamine oxidase-B inhibitors [21,22,23]. In some cases, stereotactic surgery is performed to lesion parts of the globus pallidus or the STN, which are overactive in PD. During the last two decades, stereotactic lesions have been replaced more and more by deep brain stimulation, which downregulates the hyperactivity of the GPi or STN by high frequent electric stimulation through implanted electrodes in these brain regions. All mentioned therapies are hampered by partly severe side effects [24,25]. Especially, the L-DOPA therapy improves the PD symptoms initially, but after some years of good therapeutic results, the so-called “honeymoon phase”, patients develop more and more dyskinesias under L-DOPA influence and the therapeutic effect decreases.

Gene therapy strategies and cell replacement therapies still have experimental character and are viewed critically for ethical reasons [26,27,28,29,30].

Since several decades another potent concept of treatment of PD-associated motor symptoms is the treatment of the striatal hypercholinism, caused by the hyperactivity of cholinergic interneurons in the CPu [31,32]. Anticholinergic drugs are given to counteract the increased content of acetylcholine in the CPu. The most common drug is biperiden, a blocker of cholinergic receptors. However, due to many harmful secondary events, prescription of anticholinergics is restricted. Anticholinergics provide good antiparkinsonian motor results, but, administered systemically, their therapeutic actions are accompanied by plenty of central and peripheral severe side effects (mostly due to blocking of the already affected parasympathetic vagal system). Central effects are tiredness, vertigo, hallucinations, memory disorders and confusion; peripheral effects besides muscle weakness and ache are mydriasis, accommodation problems, raised intraocular pressure, dry mouth and dry eyes, inflammation of the salivary glands, diminished peristalsis of the esophagus and regurgitation, obstipation, micturition problems, prostate gland problems, tachycardia, and fever [5,11,13,16,19,33,34,35].

Ideally, patients should benefit from the advantages of anticholinergic therapy and, at the same time, be secured from its side effects by restricting the anticholinergic action to the hypercholinergic CPu. For this purpose, our group investigates the experimental application of Botulinum neurotoxin-A (BoNT-A) into the CPu of hemiparkinsonian rats as BoNT-A is known to block especially cholinergic signaling (Figure 1B,C).

### 1.2. Botulinum Neurotoxins/Botulinum Neurotoxin-A

Botulinum neurotoxins (BoNTs) are known to cause botulism, a severe paralysis in humans and cattle. Botulism results mostly from the consumption of wrongly stored or foul food or wrongly fermented silage in the case of cattle. BoNT poisoning is characterized by flaccid paralysis of skeletal muscles and vegetative disturbances. Symptoms are life threatening because of respiratory paralysis. Species of the anaerobic genus *Clostridium* produce different types of BoNTs. Currently, eight serotypes of BoNT (BoNT-A, -B, -C, -D, -E, -F, -G, and -H) are known, some of which are subdivided into different genetic subtypes. BoNTs are metalloproteases that can crack various elements of the vesicle fusion apparatus of the presynaptic membrane-proteins of the SNARE complex [35,36,37,38].

### 1.3. Experimental Intracerebral Applications of BoNTs in the Central Nervous System

Only a few groups have investigated the effects of different serotypes of BoNT by injection into various parts of the vertebrate central nervous system (CNS).

Hagenah et al. (1977) injected BoNT-A into the triceps surae muscle, the dorsal root and the spinal cord of cats to investigate if peripheral application of BoNT-A leads to disturbances of signal transduction to Renshaw cells and/or from Renshaw cells to Ia inhibitory interneurons. They found that only direct injection of BoNT-A into the spinal cord leads to changes in the activity of Renshaw cells, whereas injection into the periphery did not affect the activity of Renshaw cells and Ia inhibitory interneurons within the test period (33–46 h) [39].

To investigate the effect of different BoNTs on the CNS of mice in vivo, Luvisetto et al. (2003) [40] performed injections of BoNT-A and BoNT-B into the lateral cerebral ventricle of male CD1 mice. The effects on body temperature, weight and the general state of health was measured. Due to the limited number of mice that were used for their experiments, the LD_50_ value of BoNT-A and BoNT-B could not be calculated exactly, but was extrapolated within a range of 0.5–1.0 × 10^−6^ mg/kg body weight for both serotypes [40]. Moreover, the effect of intraventricular injection of BoNT-A or BoNT-B on locomotor behavior after systemic cholinergic or anticholinergic medication and cognitive abilities were studied [41]. Intracerebroventricular injection of BoNT-A or BoNT-B did not lead to impairment of associative learning, but it impaired the ability of discrimination of new objects. Furthermore, it enhanced the stimulant effect of scopolamine on motor behavior and the depressive effect on locomotion of oxotremorine was reduced [41]. The same group examined whether it was possible to alter the sensibility for formalin-induced pain in CD1 mice by subcutaneous or ventricle injections of BoNT-A and BoNT-B [42]. An antinociceptive effect of subcutaneously applied BoNT-A and BoNT-B and of intracerebroventricular applied BoNT-A on formalin-induced pain was found [42].

The antinociceptive potential of BoNT-A was also tested by Chaddock et al. (2004) [43]. They replaced the C-terminal domain of the BoNT-A heavy chain by *Erythrina cristagalli* lectin. The modified BoNT was injected into the dorsal horn of rat spinal cord to inhibit the local release of substance P and glycine. Electrophysiological measurements revealed a reduced C-fiber-evoked activity and reduced C-fiber-evoked responses to pain after intrathecal application of the altered BoNT conjugate. Furthermore, an analgesic effect in rats was observed [43].

Caleo et al. (2007) [44] investigated the role of neuronal activity for visual cortex maturation by temporal blockade of synaptic signal transduction. For that purpose, they performed unilateral injection of BoNT-E directly into the visual cortex of rats. They assessed the functional maturation of the rat binocular striate cortex at P34–P36 and found that unilateral inhibition of the visual cortex by BoNT-E at P35 led to permanent impairment not only of the treated but also of the contralateral visual cortex.

Evidence for microtubule-dependent anterograde and retrograde axonal transport of BoNT-A in the CNS was given by experiments of Antonucci et al. (2008). They injected unilaterally BoNT-A into the hippocampus of mice. Immunohistochemistry for the cleavage product of BoNT-A, cleaved SNAP-25, was performed. They detected immunoreactivity for cleaved SNAP-25 in the left (BoNT-A-injected) hippocampus and also in the right untreated hippocampus. Furthermore, Antonucci et al. (2008) injected BoNT-A into the superior colliculus of Sprague Dawley rats. Afterwards, they detected a truncation of SNAP-25 by BoNT-A not only in the superior colliculus but also in the contralateral retina and the ipsilateral visual cortex. Thereby they proved the axonal transport of BoNT-A from the left to the right hippocampus and from the superior colliculus to the contralateral retina and the ipsilateral visual cortex. Moreover, the transport of BoNT-A from the periphery into the CNS was demonstrated by Antonucci et al. performing unilateral injections of BoNT-A into the hippocampus and superior colliculus of Sprague Dawley rats [45]. This study was supplemented by a report of the same group, in which the anterograde and transcytotic transport of BoNT-A after intraocular injection in Long-Evan rats was shown [46].

To establish an animal model of dementia, Ando et al. (2002) blocked cholinergic signal transmission in the entorhinal cortex of rats by direct injection of BoNT-A into the entorhinal cortex [47]. Cognition tests revealed that bilateral injection of BoNT-A causes more cognitive deficits than a unilateral injection [47]. Lackovic et al. (2009) showed that even a single intracerebroventricular injection of BoNT-A in rats leads to long lasting cognitive impairments in these animals [48].

Injections of BoNT-A into the dorsomedial and dorsolateral striatum of CD1 mice to inhibit neurotransmitter release were studied by De Leonibus et al. (2011). The role of these brain regions for learning and training orientation abilities in a plus maze test was evaluated. The majority of mice develop a response strategy after long lasting training to find an arm, which is baited with food reward. That means, these mice learned to make a particular body turn to find the right arm. A minority of mice normally develop a place strategy to find the right arm, which means that the mice use spatial information to orient themselves in the plus maze. After a certain training period, the food reward was denaturized/spoiled by addition of lithium chloride (reward devaluation). After reward devaluation, the mice that had developed a response strategy to find the baited arm, shifted to a place strategy to find the right arm. Remarkably, mice that were injected with BoNT-A into the dorsomedial CPu maintained the response strategy after reward devaluation and did not switch to place strategy. The authors speculate, that the dorsomedial CPu may be important for linking spatial and outcome-related information. The injection of BoNT-A into the dorsolateral CPu of mice, seemed to promote their ability to switch from response strategy to place strategy in finding the baited arm [49].

As an experimental therapy option of epilepsy the injection of BoNT-E into the hippocampus of rats was tested by Costantin et al. (2005) [50] and Antonucci et al. (2009) [51] in the kainic acid induced mesial temporal lobe epilepsy model. Since BoNT-E can block the release of glutamate, these authors demonstrated the possibility to prevent epileptogenic hyperexcitations. Gasior et al. (2013) took up previous results and injected 1, 3.2 or 10 ng BoNT-A or BoNT-B into the amygdala of Sprague-Dawley rats to attenuate seizures, which were provoked by electric stimulation of the amygdala. They showed that BoNT-B prevented or attenuated seizures in rats more than BoNT-A. No inflammation or neuronal degeneration after BoNT application was detected, but the authors saw minimal damage around the injection cannula and the stimulation electrode, respectively [52].

Due to the ability to prohibit glutamatergic signal transmission Antonucci et al. (2010) tested the neuroprotective effect of BoNT-E in a rat stroke model. They wanted to prevent an excessive release of cytotoxic glutamate that usually follows an ischemic event. BoNT-E injection 20 min after induction of ischemia into the hippocampus led to an enhanced survival of neurons [53].

## 2. Intrastriatal BoNT-A Application

### 2.1. Dose Finding

The group of Wree et al. (2011) reported for the first time the experimental injection of BoNT-A into the CPu of rats [54] and mice [55] to cure cholinergic motor symptoms in a PD animal model.

**Figure 2:**
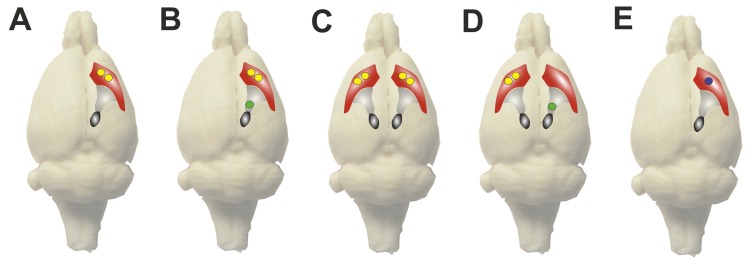
Injection sides of BoNT-A and 6-OHDA in different studies. The figure depicts the injection sites of 6-OHDA and BoNT-A of the studies reviewed in the text. The single images (**A**–**E**) show a montage of a photo of a frozen rat brain and the stylized CPu (red), the medial forebrain bundle (light grey) and the SNpc (dark grey/black). Yellow circles symbolize injection sites of BoNT-A or vehicle substance respectively. Green circles symbolize injection sites of 6-OHDA or vehicle substance respectively. The blue circle symbolizes an injection site, which is used first for the injection of 6-OHDA and later for BoNT-A. (**A**) A unilateral injection of BoNT-A into the right CPu of healthy rats such as described in Wree et al. (2011), Holzmann et al. (2012), Antipova et al. (2013), (Mehlan et al. (2016) and Mann et al. (2018a) [54,58,59,60,61]. (**B**) A unilateral injection of BoNT-A in rats that were injected with 6-OHDA into the right medial forebrain bundle one month before. BoNT-A injection was performed ipsilateral to the lesion side. This procedure was described by Wree et al. (2011), Antipova et al. (2013, 2017), Wedekind et al. (2017) and Mann et al. (2018a, b) [54,58,59,62,63,64]. (**C**) The bilateral injection of BoNT-A into the CPu of healthy rats reported Holzmann et al. (2012) is schematized here [61]. (**D**) The injection of BoNT-A into the CPu contralateral to the 6-OHDA lesion side reported in Antipova et al. (2017) is depicted here [62]. (**E**) Injection of 6-OHDA and the whole dose of BoNT-A into one injection site. This course of action was performed by Itaukra et al. (2014) [65,66].

The optimal dosage of intrastriatally applied BoNT-A for experimental hemi-PD-treatment in rats was based on information in reports on the LD_50_ and the intracerebral BoNT-A injections [44,45,49,56,57]. It was found that an amount up to 2 ng BoNT-A per CPu is tolerated in Wistar rats and that 5 ng led to death of the animals [54] (Figure 2A). In C57BL/6 mice, 25–50 pg BoNT-A per CPu were well tolerated and did not lead to health problems [55].

### 2.2. Experimental Application in Rats

#### 2.2.1. Unilateral Application in Naїve Rats

Wree and colleagues reported for the first time that a unilateral intrastriatal injection (Figure 2A) of 1–2 ng BoNT-A into the right CPu of Wistar rats led to an apomorphine-induced turning behavior of 2–3 rotations per minute, directed ipsilateral to the injected side [54,58]. This effect was observed from two weeks up to three months after BoNT-A application and vanished afterwards. The unilateral injection of 100 pg BoNT-A did not provoke measurable apomorphine-induced rotation behavior.

**Figure 3:**
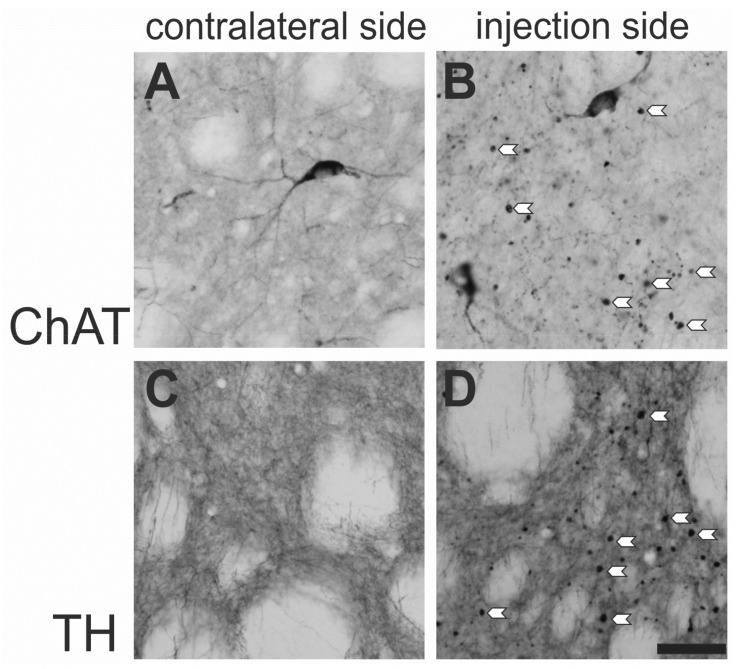
BoNT-A-induced varicosities (BiVs). Immunohistochemical reactions for choline acetyl transferase (ChAT) (**A**,**B**) and tyrosine hydroxylase (TH) (**C**,**D**) of the CPu. The rat was injected with 2 ng BoNT-A into the right CPu, the left CPu remained untreated. Panel (**A**,**C**) are from the left CPu and (**B**,**D**) from the BoNT-A injected CPu. White arrowheads mark some ChAT-positive (**B**) and TH-positive (**D**) BiVs exemplary, both only detectable in the BoNT-A-injected hemisphere. The scale bar corresponds to 50 µm (**A**–**D**).

The same group also described swellings of neuronal processes in the CPu after intrastriatal injection of BoNT-A [54]. Swellings were only found along neuronal branches in the BoNT-A-treated CPu, had a diameter between 2 and 9 µm, and were never found in the contralateral CPu (Figure 3). These swellings were termed BoNT-A-induced varicosities (BiVs). They were visible on cholinergic and catecholaminergic fibers in the CPu. Their numeric density was shown to be dose-dependent, since rats treated with 100 pg BoNT-A per CPu possessed the lowest numeric density of BiVs, rats treated with 2 ng BoNT-A the highest density (Table 1). Swellings on neuronal branches were not seen in immunohistochemical stainings for other transmitter systems and their neuronal processes. An electron microscopic analysis of tyrosine hydroxylase (TH)-immunoreactive BiVs identified them as large axonal dilations filled with vesicles and some mitochondria. A synaptic character of these swellings such as in synapses en passant was not found [54].

In a stereological analysis of BoNT-A-treated brains after intrastriatal injection of 100 pg or 1 ng BoNT-A, changes in neither the striatal volume nor the number of cholinergic interneurons therein were detected [54]. Furthermore, injection of 1 ng BoNT-A into the right CPu of rats did not change the total number of striatal neurons as compared with the uninjected contralateral CPu [59]. Immunohistochemical reactions for microglia (Iba1) and astrocytes (GFAP) showed that BoNT-A injection into the CPu does not lead to inflammatory reactions [59].

In the work of Mehlan et al. (2016) the effects of unilateral intrastriatal injection of 1 ng BoNT-A on the histology of the CPu were investigated more precisely [60]. The temporal changes induced by BoNT-A injection from two weeks up to one year after surgery were evaluated. Counting of ChAT-positive neurons, counting of TH- and ChAT-positive BiVs and measurements of their volumes were done two weeks and 1, 3, 6, 9 and 12 months after unilateral BoNT injection. During the whole experiment, no significant differences in the number of cholinergic interneurons between the right BoNT-A-treated CPu and the left untreated CPu were measured. A mean of about 30,000 cholinergic interneurons per CPu was determined. Mehlan et al. found the numeric density and the volume of ChAT-positive as well as TH-positive BiVs to underlie temporal dynamics. Therefore, the numeric density of BiVs decreased steadily over time, the volume of single BiVs increased within one year. They found 9085–11,582 ChAT-positive BiVs per mm^3^ with a single volume ranging from 18.96 µm^3^ per BiV after two weeks up to 51.49 µm^3^ per BiV after one year, and 21,445–47,125 TH-positive BiVs per mm^3^ with a single volume from 15.35 µm^3^ per BiV after two weeks to 35.97 µm^3^ per BiV after one year. It was shown that BiVs are organized like stringed bead chains on single axons. The authors speculate that single BiVs at a neuronal branch consist of accumulations of intra-axonal vesicles and that several small BiVs merge over time to fewer but larger BiVs. This would explain the decreasing number of BiVs and the increasing volume of the remaining BiVs over [60] (Table 1).

In immunohistochemical reactions for the cleavage product of BoNT-A, i.e., cleaved SNAP-25, the main specific immunoreactivity remained limited on the injected side. However, slight reactivity for cleaved SNAP-25 was also detected in the ipsilateral motor and somatosensory cortex as well as in the ipsilateral hypothalamus [61].

#### 2.2.2. Unilateral Application of BoNT-A in the Hemiparkinsonian 6-OHDA Rat Model

The 6-hydroxydopamine (6-OHDA) induced unilateral model of hemi-PD in rodents is well established and often used for therapy studies on motor deficits in experimental PD [67,68,69,70,71,72]. The group of Wree et al. performed unilateral injections of 6-OHDA into the right medial forebrain bundle [54,58,59,62,63,64] to provoke a maximum (~90% or more) demise of dopaminergic neurons in the right SNpc [67,73] (Figure 2B). In a more incomplete degeneration model of the SNpc, Itakura et al. injected 6-OHDA into the CPu [65,66] (Figure 2E).

The two groups differently confirmed the success of the 6-OHDA lesion. Wree et al. (2011), Antipova et al. (2013, 2017), Mann et al. (2018a, b) and Wedekind et al. (2017) performed apomorphine-induced rotation tests one month after the lesion. Rats were considered to be hemiparkinsonian when they showed at least four apomorphine-induced rotations per minute directed contralateral to the lesion side [54,58,59,62,63,64]. Itakura et al. (2014a, b) performed a methamphetamine-induced rotation test seven days after the striatal 6-OHDA injection and considered rats as successfully lesioned when they showed at least 1.66 rotations per minute (“50–200 turns within 30 min”) [65,66].

##### BoNT-A Application Ipsilateral to the 6-OHDA-Lesioned Side

• Apomorphine-induced rotation test

Wree and colleagues described a significant suppression of the pathological apomorphine-induced rotation rate in hemi-PD rats up to 6 months in BoNT-A-treated rats. Within 12 months after the BoNT-A injection, the apomorphine-induced turning rate gradually increased again [54,58,59,62,63,64]. A sham treatment with the vehicle solution without BoNT-A had no significant effect on the apomorphine-induced turning behavior in hemi-PD rats. The authors mentioned that the observed effect of BoNT-A in the striatum on the apomorphine-induced rotation rate lasted longer than the known BoNT-A-induced effects in the periphery like paralysis of skeletal muscles or inactivation on the salivary glands. They speculated that differences in the vesicle fusion apparatus of cholinergic synapses in the peripheral nervous system and the CNS might exist [54,58,59,62,63,64] (Table 1).

• Amphetamine-induced rotation test

In hemiparkinsonian rats, the systemic application of d-amphetamine sulfate led to a turning behavior which was directed ipsilateral to the 6-OHDA lesion side and by this exactly contralateral to the apomorphine-induced turnings. It was found that this amphetamine-induced turning rate was tendentially [59,63] or even significantly [62] increased after intrastriatal injections of 1 ng BoNT-A. The application of 2 ng BoNT-A led to a significant increase of the turning behavior under amphetamine influence three months after BoNT-A injection [59] (Table 1).

• None drug-induced tests for motor abilities

BoNT-A injections which were performed into the CPu ipsilateral to the 6-OHDA lesion led to a readjustment of the spontaneous forelimb use in the cylinder tests described by Schallert and Tillerson (2000) [74]. Especially, high doses of 2 ng BoNT-A significantly improved the asymmetry in forelimb use in hemi-PD rats, whereas doses of 1 ng BoNT-A had no significant effect [54,59,62].

The asymmetry of forced motor abilities of the forelimbs of hemi-PD rats after BoNT-A treatment was evaluated by the stepping test [62]. Within most time points after injections, BoNT-A-treated rats and sham-treated rats did not differ in their performance in the stepping test [62].

Accelerod and open field tests revealed that BoNT-A treatment of hemi-PD rats had no consequences on balance, motor coordination and spontaneous motor activity in comparison with hemi-PD rats which were sham-treated or sham-lesioned and sham-treated rats [59,62].

Tests for unilateral deficits in sensorimotor integration or unilateral neglect in rats by corridor task revealed that 6-OHDA lesion of the right SNpc led to a neglect of the left body side, and an ipsilateral BoNT-A injection into the CPu did not improve sensorimotor integration [62] (Table 1).

##### Application of BoNT-A Contralateral to the 6-OHDA-Lesioned Side

For a more profound analysis of the working hypothesis that the hypercholinism in the CPu ipsilateral to the 6-OHDA lesion can be cured by BoNT-A injection [54], the experimental design was extended by establishing a further experimental group, which was 6-OHDA-lesioned on the right side and treated with BoNT-A in the CPu of the unlesioned (contralateral) side [62] (Figure 2D). Interestingly, the BoNT-A injection contralateral to the lesioned side two weeks after surgery led to a significant increase of the apomorphine-induced turning rate, but additional two weeks later (four weeks after BoNT-A) the apomorphine-induced turning rate was significantly decreased again. The amphetamine-induced rotation behavior was tentatively decreased two weeks after contralateral BoNT-A injection [62].

Remarkably, contralateral application of 1 ng BoNT-A led to a significant readjustment of left and right forelimb usage in hemi-PD rats. This effect decreased again during the following weeks. In contrast to the ipsilateral BoNT-A application, an injection of BoNT-A contralateral to the 6-OHDA lesion improved the motor abilities of both forelimbs. Therefore, significantly more adjusting steps were counted for both forelimbs compared to sham-treated hemi-PD rats [62].

BoNT-A application into the CPu contralateral to the lesioned side led to a significant reduction of neglect of the left side. This effect did not occur in rats, which were BoNT-A-treated ipsilateral to the 6-OHDA lesion. The contralaterally injected hemi-PD rats showed a significant readjustment of left and right retrievals [62].

According to the ipsilateral BoNT-A injection, BoNT-A injection contralateral to the 6-OHDA-lesioned side did not lead to differences in spontaneous locomotor behavior compared with sham-treated animals (Table 1).

#### 2.2.3. Bilateral BoNT-A Application in Rats and Consequences for Motor Behavior and Cognition

Pursuing the objective to establish a new anticholinergic therapy poor in side effects, it has to be examined if intrastriatal BoNT-A application affects other cholinergic brain regions, which are important for motor or cognitive abilities, like the pedunculopontine nucleus and the nucleus basalis of Meynert [75,76,77]. Therefore, Holzmann et al. (2012) performed investigations on naïve Wistar rats treated with bilateral intrastriatal BoNT-A injection [61] (Figure 2C) (Table 1).

It was shown that the bilateral intrastriatal BoNT-A injection in naïve rats impaired their performance on the accelerod testing balance and motor coordination. Moreover, the spontaneous motor activity in the open field was significantly decreased compared with sham-treated and naïve rats. No changes in body temperature within four weeks after BoNT-A application were detected. In the open field and the elevated plus maze tests, evidence for reduced anxiety of bilaterally BoNT-A-treated rats was found. In bilaterally BoNT-A-treated rats no impairments of the reference memory studied with the Morris water maze test was detected. In the radial maze test, however, hints for a worsening of the working memory of bilaterally BoNT-A-treated rats were observed. However, it could not be excluded that the changes in working memory were due to the surgical procedure, because the differences were significant between the results of BoNT-A-treated and naive rats only, but not between BoNT-A- and sham-treated rats [61].

#### 2.2.4. In Vitro Receptor Autoradiography and Positron Emission Tomography

It has been speculated that the beneficial effect of unilateral BoNT-A injection into the CPu of rats ipsilateral to a 6-OHDA lesion on apomorphine-induced rotation rate can be explained by changes of the density of transmitter receptors in the CPu. It is well known that a 6-OHDA lesion of the SNpc leads to an upregulation of the D_2_ receptor density in the ipsilateral CPu [78].

In vitro receptor autoradiography in hemi-PD rats, which were treated intrastriatally with 1 ng BoNT-A or vehicle substance was performed by Mann et al. (2018a, b). They investigated the effect of this treatment especially on D_1_ and D_2_/D_3_ receptors. D_1_ receptors where labeled with [^3^H]-SCH 23,390 and D_2_/D_3_ receptors with [^3^H]-Fallypride. The first group consisted of untreated rats, the second group of hemi-PD rats, the third group of naïve rats treated unilaterally with 1 ng BoNT-A only, the fourth group were hemi-PD rats treated with BoNT-A, and the fifth group were hemi-PD rats that had received the vehicle substance of BoNT-A into the right CPu. The animals were studied 1–9 months after the last surgery. It was shown that intrastriatally applied BoNT-A reduced the density of D_2_/D_3_ receptors in naїve rats. 6-OHDA lesion of the right SNpc led to an increase of the D_2_/D_3_ receptor concentration in the right CPu. Hemi-PD rats injected with BoNT-A showed a significant readjustment of the right D_2_/D_3_ receptor density. Moreover, significant linear correlation between the relative difference of interhemispheric D_2_/D_3_ receptor density in the CPu and the apomorphine-induced rotation rate of hemi-PD rats was detected [58].

The density of D_1_ receptors was not changed by BoNT-A injection into hemi-PD rats or naïve rats [58].

Additionally, Mann et al. (2018b) investigated longitudinally D_2_/D_3_ receptor binding potential in hemi-PD rats and sham-lesioned rats, which were either treated with 1 ng BoNT-A or with vehicle by application of the radioligand [^18^F] Fallypride and PET analysis in vivo. Measurements were done in the same rats 4, 12 and 24 weeks after BoNT-A injection. In contrast to the studies of Wedekind et al. (2017), Mann et al. (2018a, b) performed additional magnetic resonance imaging to improve data analysis and established another experimental group of sham-lesioned and sham-BoNT-A-injected rats [58,63]. Their results are in line with previous receptor autoradiography studies and the PET/-CT results of Wedekind et al. (2017) [64]. Thus, the D_2_/D_3_ receptor binding potential was significantly increased in the right CPu by 6-OHDA lesion of the right SNpc. BoNT-A injection into the right CPu reduced this increased D_2_/D_3_ receptor density and, at the same time, reduced the apomorphine-induced turning rate [58,63,64]. The difference between D_2_/D_3_ receptor densities in the left CPu and right CPu correlated with the apomorphine-induced turning rate [58] (Table 1).

### 2.3. Experimental Intrastriatal BoNT-A Application in Mice

Hawlitschka et al. (2017) evaluated the effect of unilateral injection of BoNT-A into the right CPu of naїve C57BL/6-mice [55]. Doses of 25 pg, 50 pg, 100 pg and 200 pg per CPu were injected to study the dose-dependent effect of BoNT-A. The mice were sacrificed 1, 3, 6 and 9 months after the BoNT-A injections to examine the possible temporal changes in the histology of the BoNT-A-treated brains. Body weights and brain weights were examined and immunohistochemistry for ChAT and TH performed, followed by stereological analysis of the brains. Injections of 25 pg did not lead to differences in the body weight between BoNT-A-treated and sham-treated mice. Mice that received higher dosages of BoNT-A (100 pg or 200 pg) had a higher body weight than mice, which received 25 pg or 50 pg BoNT-A. Mice that received 50 pg or 100 pg BoNT-A had significantly lighter brains than those which received 25 pg. The injection of 25 pg BoNT-A or vehicle substance did not lead to differences between the right (injected) CPu and the left (untreated) CPu. The number of cholinergic interneurons in the right (injected) and left (untreated) CPu of all animals was counted. Animals never differed in the number of cholinergic interneurons between both CPu. No differences in the number of cholinergic interneurons between sham-treated mice and BoNT-A-treated mice were found. Moreover, the application of high doses of 200 pg of BoNT-A did not lead to a loss of cholinergic interneurons.

According to the results in rats ChAT-positive BiVs were found in the BoNT-A-treated CPu, in mouse sections stained for cholinergic structures, but never in the contralateral CPu. As in rats, the numeric density of the cholinergic BiVs decreased in mice over time. The maximal numeric densities of cholinergic BiVs were found for a BoNT-A dose of 50 pg per CPu whereas in higher and lower dosages the numeric density was a slightly smaller. The volume of single BiVs was time-dependent, since the smallest BiVs were found one month after injection (5.87 µm^3^) and the biggest ones after nine months (13.7 µm^3^). The volumes of single BiVs were also dose-dependent. Therefore, the median BiV volumes after six months survival time were higher for mice which received 100 pg (21.66 µm^3^) or 200 pg (20.82 µm^3^) than those of mice which received 25 pg (11.42 µm^3^) or 50 pg (11.62 µm^3^).

Remarkably, in contrast to rats, catecholaminergic BiVs were never found in BoNT-A-treated CPu sections of mice [55]. It has been speculated that interspecific differences in synaptic vesicle glycoprotein 2C (SV2C) subtypes and their BoNT-A binding affinity may be responsible for the results for mice contray to the results for rats. Probably the SV2C subtype of dopaminergic axon terminals of C57BL/6-mice does not allow BoNT-A to bind there and BoNT-A cannot enter these axon terminals whereupon no TH-positive BiVs in mice could be formed [35,55,79].

## 3. Experimental Comparison of Intrastriatal Application BoNT-A Subtype 1 and BoNT-A Subtype 2

### 3.1. Main Findings

Itakura et al. (2014a, b) took up the thesis of Wree et al. (2011) and Antipova et al. (2013) that intrastriatally applied BoNT-A could serve as an experimental treatment option of PD symptoms [54,59,65,66]. Itakura et al. compared the effects of BoNT-A subtype 1 and BoNT-A subtype 2 in concentrations of 0.1, 0.5 and 1 ng per CPu on hemi-PD rats (Figure 2E). They found that BoNT-A subtype 1 and subtype 2 each suppressed methamphetamine-induced rotation behavior, but BoNT-A subtype 2 abolished rotation behavior in low dosages of 0.1 and 0.5 ng per CPu significantly, whereas BoNT-A subtype 1 reduced turning rate significantly only when given in a dose of 1 ng. Analysis of immunofluorescence staining for BoNT-A-cleaved SNAP-25 in BoNT-A-injected CPu revealed that BoNT-A subtype 2 cleaved SNAP-25 more efficiently than BoNT-A subtype 1 [65]. Itakura et al. (2014b) found a significant loss of body weight in rats intrastriatally treated with BoNT-A subtype 1, but not in rats treated with subtype 2 [66]. Immunofluorescence staining for BoNT-A-cleaved SNAP-25 revealed that the effect of BoNT-A subtype 2 is limited to the injected CPu, whereas in brains injected with BoNT-A subtype 1 into the right CPu, cleaved SNAP-25 was detectable in the ipsilateral as well as in the contralateral CPu. Therefore, the authors suggested that BoNT-A subtype 2 action was limited to the injection area, but BoNT-A subtype 1 likely diffused into the contralateral CPu or got there by axonal transport [66]. Subsequently, Itakura et al. concluded that BoNT-A subtype 2 should be more efficient in treatment of PD symptoms than BoNT-A subtype 1, and that BoNT-A subtype 2 seemed safer for pharmacological utilization in the CPu.

### 3.2. Discussion of Itakura’s Findings and Integration with Other Results

It is remarkable that Itakura et al. (2014a) observed a reduction of the methamphetamine-induced rotation rate in hemi-PD rats after intrastriatal BoNT-A injection [65], whereas Antipova et al. (2013, 2017) and Mann et al. (2018b) found that intrastriatal BoNT-A injection led to a tentative or even significant increase of amphetamine-induced rotation rates [59,62,63]. This finding is astonishing because methamphetamine and d-amphetamine pharmacologically act quite similar: they induce reverse transport of dopamine by the dopamine transporter and block the reuptake of dopamine [80,81], thereby increasing the concentration of dopamine in the synaptic cleft. Explanations for these divergent findings may be found in differences of the experimental concepts: Itakura et al. (2014a, b) investigated the effect of intrastriatal BoNT-A-application in Sprague-Dawley rats [65,66], whereas Wree and colleagues and Wedekind et al. (2017) performed their studies on Wistar rats [54,58,59,62,63,64]. Seemingly, the main reason for the differences in the results of Itakura et al. and our group is that Wree and colleagues performed a unilateral injection of 6-OHDA into the right medial forebrain bundle [54,58,59,62,63,64] (Figure 2B,D), but Itakura et al. into the right CPu [65,66] (Figure 2E). 6-OHDA injections into the medial forebrain bundle provoke a maximum (~90%) demise of dopaminergic neurons of the substantia nigra pars compacta [69,73]. It is well known, that application of 6-OHDA into the MFB leads to a more complete and faster loss of dopaminergic neurons in the ipsilateral SNpc than an injection of 6-OHDA into the CPu [68]. Thus, the loss of dopaminergic terminals in the CPu of the rats in the studies of Wree et al. (2011), Antipova et al. (2013, 2017), (2018a, b) and Wedekind et al. (2017) was nearly complete, whereas the loss of striatal dopaminergic axons in the rats studied by Itakura et al. (2014 a, b) was probably only partial. In the case of a partial lesion or a transient loss of dopaminergic fibers in the CPu methamphetamine could enhance the dopamine concentration in the right CPu. However, in brains with a total dopaminergic deafferentation of the right CPu, amphetamine could enhance the dopamine concentration only in the left CPu but not in the right one, because there are almost no axon endings which could release dopamine. Hawlitschka et al. (2013), Wedekind et al. (2017) and Mann et al. (2018a, b) demonstrated the success of the right side 6-OHDA lesion by immunohistochemistry for TH or Nissl stainings of the SNpc as well as the CPu [58,63,64,82]. Unfortunately, Itakura et al. (2014a, b) did not present such a validation of successful 6-OHDA lesion and rated the success of the lesion by methamphetamine-induced turning rate only [65,66].

Moreover, both groups used different doses of methamphetamine or amphetamine, respectively, injected intraperitoneally prior the rotation tests. Itakura et al. (2014a, b) injected 2 mg/kg methamphetamine [65,66], whereas Antipova et al. (2013, 2017) and Mann et al. (2018b) injected 2.5 mg/kg amphetamine [59,62,63].

Furthermore, Wree et al. (2011), Antipova et al. (2013, 2017), Mann et al. (2018a, b) and Wedekind et al. (2017) approved the success of the 6-OHDA lesion by apomorphine-induced rotation test and performed amphetamine-induced rotation tests as a further tool to examine the effect of BoNT-A treatment on basal ganglia circuitry.

Itakura et al. performed the first rotation tests seven days after 6-OHDA lesion, whereas Wree et al. (2011), Antipova et al. (2013, 2017), Mann et al. (2018a, b) and Wedekind et al. (2017) carried out the first drug-induced rotation tests four weeks after the lesion. The injection of the BoNT-A subtypes or vehicle was carried out two weeks after the 6-OHDA lesion by Itakura et al. [65,66], whereas others led the animals recover for at least one month between the surgeries [54,58,59,62,63,64].

Moreover, differences occurred in the application of BoNT-A. The whole amount of BoNT-A which an animal received within one treatment, was injected in one site only by Itakura et al. (2014a, b) [65,66] (Figure 2E), whereas Wree et al. (2011), Antipova et al. (2013, 2017), Mann et al. (2018a, b) and Wedekind et al. (2017) divided the respective volume of BoNT-A solution and injected it into two injection sites into the CPu [54,58,59,62,63,64] (Figure 2A–D). With the injection of BoNT-A into two coordinates Wree and colleagues wanted to ensure that a wide part of the CPu was influenced by BoNT-A.

## 4. Discussion of the BoNT Doses

It is remarkable that the intracerebrally injected doses of BonT-A in rats and mice exceed the LD_50_. It has been reported that the LD_50_ of BoNT-A for mice is between 0.25 and 1.15 ng/kg [36]. At the time of intrastriatal BoNT-A injection, mice had a mean body weight of about 20 g resulting in an calculative LD_50_ between 5 pg to 23 pg BoNT-A per mouse. Rats weighted on average 300 g at the time of BoNT-A injection. Extrapolating the LD_50_ values of mice results in an LD_50_ between 75 pg and 345 pg BoNT-A per rat. In mice, intrastriatal BoNT-A applications were performed with doses of 25 pg up to 200 pg and in rats doses between 100 pg and 5 ng were injected, an enhanced mortality of rats being detected only at a dosage of 5 ng. Two ng BoNT-A were well tolerated by rats, and also mice did not show severe health problems after injections of 200 pg. It has to be considered that in toxicity studies and mouse bioassays BoNT solutions were injected intraperitoneally. Thus, a quick uptake of the toxin by the peritoneum and by the gastrocolic omentum occurs. Following intraperitoneal injection BoNT-A acts systemically and animals die mostly caused of paralysis of the respiratory muscles after application of a lethal dose. One can suppose, that by injection of BoNT-A into the parenchyma of the brain as into the CPu, BoNT-A is trapped by the blood–brain barrier and cannot act in the periphery.

## 5. Future Prospects

The rationale for local BoNT-A injection into the striatum of hemi-PD rats is to decrease the release of ACh by disinhibited tonically active cholinergic interneurons. Here, hypercholinism seems to be responsible for a disturbed network of the basal ganglia and consecutive motor and behavioral dysfunction [8,83,84]. Current therapeutic approaches in PD among others use anticholinergic drugs [85,86]. However, anticholinergic medication, e.g., the piperidine derivative biperiden (Akineton^®^), is compromised by adverse side effects such as dyskinesia and anticholinergic syndrome due to systemic drug application [16,19,33,87]. The beneficial effect of direct injection of BoNT-A into the CPu of hemi-PD rats lasted for 3–6 months [54,59,60,61,62,82]. Second, it is of high interest to test whether repeated intrastriatal BoNT-A injections are generally possible and able to improve motor behavior in hemi-PD rats for a longer period resembling clinical practice for BoNT-A treatments in man [88,89,90,91]. Third, we will extend studies of intrastriatal BoNT-A injections to naïve mice. In experimental Parkinson research, mice with mutations or knockouts of relevant PD-associated genes are under consideration to get more insights into disease-related pathological mechanisms [92,93,94,95,96]. Interestingly, rat and mouse striata differ in morphological and behavioral reaction to BoNT-A application [55] indicating species-specific differences.

BoNT-A or other subtypes may be useful in the treatment of brain disorders in case deactivation of local brain activity is needed [59]. As BoNTs act for a limited time the reversibility is an advantage. Optimistically, in the future and after experiments in primates, BoNTs might be used in clinical application as an effective and individually-tailored “chemical neurosurgical approach” [51,52].

## Figures and Tables

**Table 1:** The table summarizes and compares characteristic results of reports concerning intrastriatal BoNT-A injection. Grey filled area means that no data are evaluable. (↓) in the column “BoNT-A influence on Drug-induced turning rate” symbolizes a decreased rotation rate and in the particular cases of healthy rats which received BoNT-A. (↓) marks an apomorphine-induced rotation rate, which is directed ipsilateral to the injection side [54,58]. (↓) marks a reduced binding potential in the column “D2-receptor binding potential after BoNT-A”. (↑) marks an increased rotation rate in the column “BoNT-A influence on drug-induced turning rate”. (−↓) marks a tendentially but not significant decreased rotation rate. (+) marks a readjustment or improvement of motor behavior or an existence of BiVs. (++) marks a rich occurrence of BiVs. (−) marks a lack of improvements in motor abilities or a nonexistence of BiVs.

Publication	Species	6-OHDA Lesion	BoNT-A			BoNT-A Influence on Drug-Induced Turning Rate		Readjustment of Forelimb Use after Unilateral 6-OHDA Lesion	Improvement of Forelimb Akinesia (Stepping Test)		Readjustment of Lateralized Sensorimotor Integration	Numeric Density BiVs		D_2_ Receptor Binding Potential after BoNT-A
			Ipsi-Lateral	Contra-Lateral	Bi-Lateral	Apo-Morphine	Amphetamine/Meth-Amphetamine	Cylinder Test	Steps of Forelimb Ipsilateral to 6-OHDA	Steps of Forelimb Contralateral to 6-OHDA	Corridor Task	ChAT Positive	TH Positive	Receptor Autoradiography or PET/CT
Wree et al. (2010) [54]	Wistar rat	right MFB	1 ng			↓		−				+	−	
			2 ng			↓		+				++	−	
			0.1 ng			↓						+	+	
			1 ng			↓						+	+	
			2 ng			↓						++	++	
Holzmann et al. (2012) [61]	Wistar rat				1 ng									
Antipova et al. (2013) [59]	Wistar rat	right MFB	1 ng			↓	−	−						
			2 ng			↓	↑	+						
Antipova et al. (2017) [62]	Wistar rat	right MFB	1 ng			↓	↑	−	−	−	−			
				1 ng		↑	−↓	+	+	+	+			
Mehlan et al. (2016) [60]	Wistar rat		1 ng									+	+	
Hawlitschka et al. (2017) [55]	C57BL/6 mouse		0.025–0.2 ng									+	−	
Wedekind et al. (2017) [64]	Wistar rat	right MFB	1 ng			↓		−						↓
Mann et al. (2018a) [58]	Wistar rat	right MFB	1 ng			↓								↓
			1 ng			↓								↓

## Abbreviations

BiVs	Botulinum neurotoxin induced varicosities
BoNT	Botulinum neurotoxin
ChAT	choline acetyltransferase
CNS	central nervous system
CPu	caudate-putamen/striatum
GPe	globus pallidus pars externa/external globus pallidus
GABA	gamma-aminobutyric acid
GPi	globus pallidus pars interna/internal globus pallidus
LD_50_	median lethal dose
L-DOPA	L-3,4-dihydroxyphenylalanine
MSN	medium spiny neurons
PD	Parkinson’s disease
SNARE	soluble *N*-ethylmaleimide-sensitive-factor attachment receptor
SNpc	substantia nigra pars compacta
STN	subthalamic nucleus
SV2C	synaptic vesicle glycoprotein 2C
TH	tyrosine hydroxylase
VL	ventrolateral thalamic nucleus
